# Impact of Crystal Structure and Particles Shape on the Photoluminescence Intensity of CdSe/CdS Core/Shell Nanocrystals

**DOI:** 10.3389/fchem.2018.00672

**Published:** 2019-01-22

**Authors:** Lukas Ludescher, Dmity N. Dirin, Maksym V. Kovalenko, Michael Sztucki, Peter Boesecke, Rainer T. Lechner

**Affiliations:** ^1^Institute of Physics, Montanuniversitaet Leoben, Leoben, Austria; ^2^Department of Chemistry and Applied Biosciences, ETH Zurich, Zurich, Switzerland; ^3^Empa - Swiss Federal Laboratories for Materials Science and Technology, Duebendorf, Switzerland; ^4^European Synchrotron Radiation Facility, Grenoble, France

**Keywords:** core/shell nanocrystals, CdSe/CdS, photoluminescence, SAXS, WAXS

## Abstract

To study the influence of the chemical and crystalline composition of core/shell NCs on their photoluminescence (PL) the mean structural profile of a large ensemble of NCs has to be retrieved in atomic resolution. This can be achieved by retrieving the chemical profile of core/shell NCs using anomalous small angle x-ray scattering (ASAXS) in combination with the analysis of powder diffraction data recorded by wide angle x-ray scattering (WAXS). In the current synchrotron based study, we investigate CdSe/CdS core/shell NCs with different core dimensions by recording simultaneously ASAXS and WAXS spectra. The CdS shells are grown epitaxial on nominal spherical CdSe cores with core diameters from around 3.5–5.5 nm. Three different CdSe shell thicknesses are realized by depositing around 4, 6, and 8 monolayers (MLs) of CdSe. We reveal that the epitaxial core/shell structure depicts a chemical sharp interface, even after a post growth annealing step. With increasing NC diameter, however, the CdSe/CdS NCs deviate significantly from a spherical shape. Instead an elliptical particle shape with pronounced surface facets for the larger core/shell NCs is found. In combination with the powder diffraction data we could relate this anisotropic shape to a mixture of crystal phases within the CdSe core. The smallest CdSe cores exhibit a pure hexagonal wurtzite crystal structure, whereas the larger ones also possess a cubic zincblende phase fraction. This mixed crystal phase fractions lead to a non-spherical shell growth with different thicknesses along specific crystallographic directions: The long axes are terminated by basal crystal faces parallel either to the *a*- or *c*-axis, the short axes by “tilted” pyramidal planes. By combining these structural data with the measured PL quantum yield values, we can clearly connect the optical output of the NCs to their shape and to their shell thickness. Above 6 ML CdS shell-thickness no further increase of the PL can be observed, but for large aspect ratio values the PL is significantly decreased. The gained understanding of the internal crystal structure on CdSe/CdS NCs is general applicable for a precise tuning of the optical properties of crystalline core/shell NCs.

## 1. Introduction

The wet-chemical synthesis of colloidal nanocrystals (NCs) is a well established method providing highly monodisperse quantum dots (Murray et al., 2000; Sun and Xia, 2002; Kovalenko et al., 2015) with unique optical (Klimov and Bawendi, 2001; Yin and Alivisatos, 2005; Achermann et al., 2006; Talapin, 2012) or magnetic properties (Park et al., 2004; Kovalenko et al., 2007; Laurent et al., 2008; Yoo et al., 2011). The additional growth of an *epitatxial shell* around the core allows, e.g., to tune the magnetic properties (Kovalenko et al., 2007) or enhance the photoluminescence (PL) quantum yield (QY) (Hines and Guyot-Sionnest, 1996; Reiss et al., 2009). Two typical methods for growing a protective shell atop a core are: Either epitaxially on top of the initial core (Hines and Guyot-Sionnest, 1996; Reiss et al., 2009; Yarema et al., 2011), or by replacing elements in the core's shell using galvanic replacements for metallic NCs (Sun and Xia, 2002; Chen et al., 2005) or cation exchange reactions (Son et al., 2004; Pietryga et al., 2008; Kovalenko et al., 2012; Sytnyk et al., 2013; Lechner et al., 2014). Beside the chemical stabilization of the optically active core, the shell enables a significantly enhanced PL-QY due to electron-hole confinement by choosing a wider band gap material for the shell as for the core (Talapin et al., 2001; Pietryga et al., 2004; Reiss et al., 2009; Kovalenko et al., 2012; Lechner et al., 2014). This PL enhancement, however, levels off when the shell thickness exceeds a critical shell thickness. This was related to defect formation caused by the strain impact of the core on the thick shell or vice versa due to the lattice mismatch between the core and the shell (Pietryga et al., 2008; Zhao et al., 2011). Additional influence on the whole core/shell structure is found, when the core and the shell crystal structure differs. For the case where the shell is grown by cationic exchange reaction, e.g., for PbS/CdS NCs, the shell keeps in the beginning the *rocksalt* structure of the PbS core, and only with growing shell thickness the transition to the equilibrium *zincblende* phase of the CdS shell occurs (Lechner et al., 2014).

To avoid problems with different core/shell crystal structures, in this study we investigate the growth of CdSe/CdS core/shell NCs and the PL ouput of these NCs. By following the wet chemical synthesis described in Chen et al. (2013) we realize CdSe cores in the hexagonal *wurtzite* crystal structure (*P*6_3_*m*, *a* = 4.30 Å, *c* = 7.00 Å) and grow slowly CdS (*a* = 4.14 Å, *c* = 6.75 Å) epitaxially on top. Thus, core/shell NCs with a uniform crystal structure can be achieved. The lattice mismatch between core and shell of around 3.8 % will imply a certain *tensile* strain on the shell material.

**Figure 1:**
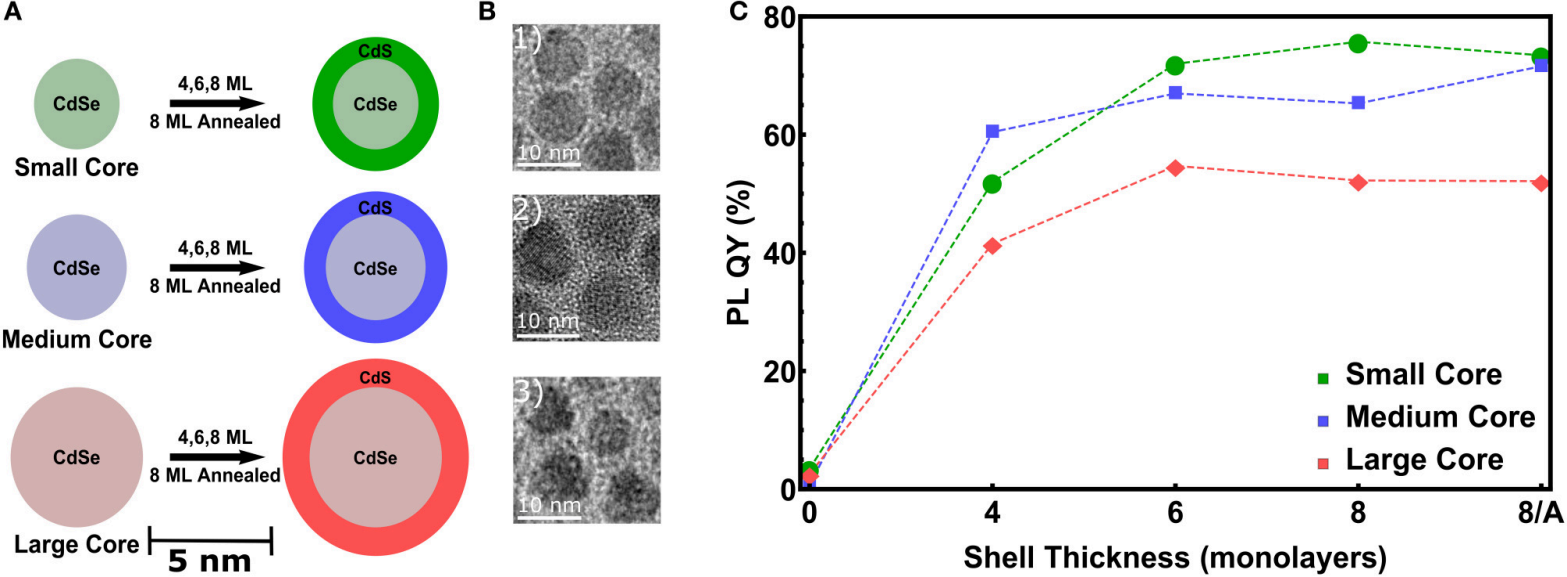
**(A)** Scheme of the used CdSe cores dimensions denoted as *small* (*D*_*core*_ = 3.6 nm), *medium* (*D*_*core*_ = 4.4 nm) and *large* (*D*_*core*_ = 5.7 nm). On top of these cores 4, 6, and 8 ML of CdS are grown. For the thickest shell an additional annealing step at 310°C is performed for the *medium* and *large* samples. **(B)** TEM images of the CdSe/CdS core/shell NCs with the thickest shell of 8 ML. **(C)** Photoluminescence quantum yield (PL-QY) as a function of the intended shell thickness for all three sample series.

To study the influence of increasing CdS shell thicknesses and of different CdSe core diameters, we have realized three series of CdSe NCs with 3.6, 4.4, and 5.7 nm diameter (*D*_*core*_), denoted as *small, medium* and *large* NCs (see Figures 1A,B). The sizes derived from TEM are 3.1, 3.9, and 4.7 nm respectively, which is significantly lower than the SAXS derived core sizes. This might be due to a potential anisotropoy of the particles, as discussed later in Section 4, which may lead to a preferred orientation of the nanocrystals in the closed packing observed in transmission electron microscopy (TEM). On these cores we intended to grow a CdS shell of 4 monolayers (MLs), 6 and 8 MLs thickness. In Figure 1C for all *three* samples series an increasing PL-QY for increasing shell thickness is evidenced. (For the single PL spectra see Figure S1) Up to 6 MLs the PL increases, above 6 MLs the QY-increase remains quite constant. If this is related to a higher stacking fault concentration within the shell, an optional annealing step can improve again the crystalline quality and hence the PL ouput (Chen et al., 2013). To prove this hypothesis for the thickest shell of 8 ML, corresponding to ~ 2.8 nm CdS, we performed for the *medium* and *large* samples an additional annealing step at 310°C, but *no* real increase in the PL-QY could be observed. The small increase after annealing for the medium sized core/shell sample is close to the error band. Remarkable, however, is the fact that the PL enhancement for the *largest* core/shell sample series is significantly lower as compared to the *small* and *medium* sized NCs (see Figure 1C).

To now relate the observed optical behavior to the chemical and crystalline profile of the core/shell NCs we have to derive the *averaged* structural parameter of the NC-batches, because also the PL data are probed from a large ensemble of individual NCs. With small angle x-ray scattering (SAXS) techniques in combination with the analysis of powder diffraction data retrieved by wide angle x-ray scattering (WAXS) we can probe the colloidal CdSe/CdS core/shell NCs directly in solution (toluene). Thus, we obtain structural information on the 3D shape of around 10^8^–10^10^ NCs, a number that is comparable to that used for optical characterizations.

The NCs' mean size and size distribution, their shape and—especially—the core diameter and shell thickness values are important parameters to explain the observed PL performance. Especially the question, if the intended ML-coverage of the CdSe core is reached for all samples homogeneously is an important question. For local sensitive microscopy techniques alone, like TEM based techniques analysing 2D images of NCs deposited on a TEM-grid, it is not easy to resolve single ML steps in the shell thickness (see Figure 1B). Even in combination with energy dispersive x-ray spectroscopy (EDX), the chemical profile of a rather small number of single core/shell NCs may be probed. Especially after the applied annealing step the question, if the core/shell NCs keep a chemically stable CdSe/CdS interface, is of importance. From the analysis of the diffraction peaks derived by WAXS we can obtain the crystalline structure and size of the NCs, but the averaged chemical profile can be revealed by anomalous SAXS (ASAXS).

Thus, synchrotron based ASAXS and WAXS measurements will be combined with TEM to reveal quantitative values of the above discussed structural parameter of the NCs. These data will be related to the measured PL output to explain the observed behavior of the PL-QY.

## 2. Chemical Synthesis of CdSe/CdS NCs

The CdSe cores were synthesized via the following procedure (Chen et al., 2013; Gollner et al., 2015). 240 mg (1.872 mmol) of CdO (Aldrich, 99.99%), 1.12 g (3.344 mmol) of octadecylphosphonic acid (ODPA, PCI synthesis, 99%) and 12 g of trioctylphosphine oxide (TOPO, Strem, 99%) were mixed in 3-neck flask and subsequently degassed for 1 h at 150°C. Afterwards, under a nitrogen flow, the temperature was elevated up to 320°C to form a colorless solution, upon which 4 mL trioctylphosphine (TOP, ABCR, 97%) was injected. The temperature was further increased to 355–390°C depending on the desired size of CdSe cores. At this point solution of 240 mg Se (Aldrich, 99.99%) in 2 mL TOP was swiftly injected. After 30–120 s the growth was stopped by rapid cooling with air flow and water bath. The resulting CdSe particles were diluted by 10 mL toluene and precipitated by 30 mL of acetone. The pellet was re-dissolved in a mixture of 8 mL hexane, 6 mL of nonanoic acid, 6 mL of octylamine and immediately precipitated by minimum amount (20–25 mL) of ethanol and the obtained powder finally dispersed in hexane. Size and concentration of CdSe cores have been estimated from absorption spectra by using previously reported calibration curves (Jasieniak et al., 2009). The epitaxial CdS shells were grown by loading the hexane solution containing 100 nmol CdSe NCs in a mixture of 3 mL 1-octadecene (ODE, Aldrich, 90%) and 3 mL oleylamine (OLAm, Aldrich, 70%). The reaction solution was degassed afterwards at room temperature under vacuum for 1 h and subsequently for 20 minutes at 120°C. The solution was again heated up to 310°C with a heating rate of 20°C /min, with nitrogen flow and magnetic stirring. Upon reaching 240°C a desired amount of shell-forming cadmium (II) oleate and octanethiol (ABCR, 98%) solutions in 6 mL ODE were injected dropwise at ca. 3 ml/h via two syringe pumps. Cadmium oleate solution in ODE was obtained by direct reaction and degassing of cadmium oxide with 2 equivalents of oleic acid in ODE at 120°C over 1 h. Amounts of shell-forming precursors were calculated from CdSe core NCs size and concentration in order to grow 8 monolayers of CdS shell. After the desired amount was injected 1 mL of degassed oleic acid (Aldrich, 90%) was introduced and the solution was further annealed at 310°C for an hour. Aliquots were extracted when shell thicknesses of around 4, 6, and 8 MLs of CdS has been achieved under the assumption that for the different core diameters the shell growth has the same efficiency (Chen et al., 2013). The resulting particles were washed with hexane/ethanol and finally dissolved in toluene, since this solvent has the best fitting x-ray transmission value within quartz capillaries with 1.5 mm in diameter as used for the planned ASAXS experiments.

## 3. Chemical Profiling Using ASAXS

In extension to SAXS, ASAXS (Stuhrmann, 1985; Goerigk et al., 2003) allows element specific contrast variation (Sztucki et al., 2010; Raghuwanshi et al., 2012) and hence the possibility to determine the chemical compositions of the core and the shell with high accuracy in an macroscopic ensemble of NCs (Lechner et al., 2014; Yarema et al., 2017). The contrast variation in ASAXS is due to the energy dependency of the atomic scattering factor *f*(*Z, E*), in particular, in the vicinity of x-ray absorption edges, where *E* is the x-ray energy and *Z* the atomic number. The non-resonant scattering term depends only on the total electron density within a material related to the overall elementary composition and the bulk density, whereas the resonant scattering term depends on *E*. By tuning the x-ray energy close to the Se-K-edge at 12.66 keV the contribution of Se (*Z* = 34*e*^−^) as a unique core element to the total scattering amplitude *f*(*Z, E*) and hence to the total scattered intensity *I*(*q, E*) can be varied significantly. The length of the scattering vector **q** is derived by *q* = 4πsinθ/λ, where λ is the x-ray wavelength and 2θ the scattering angle. From a minimum set of 3 ASAXS spectra measured at different energies we can separate independently the total electron density from the Se-electron density inside the core/shell NCs. This was achieved by applying a step-like spherical core/shell model to fit all scattering curves (for the used form factor see Equation S1 in the Supplementary Material and Lechner et al., 2014 for a detailed description of the used ASAXS method).

**Figure 2:**
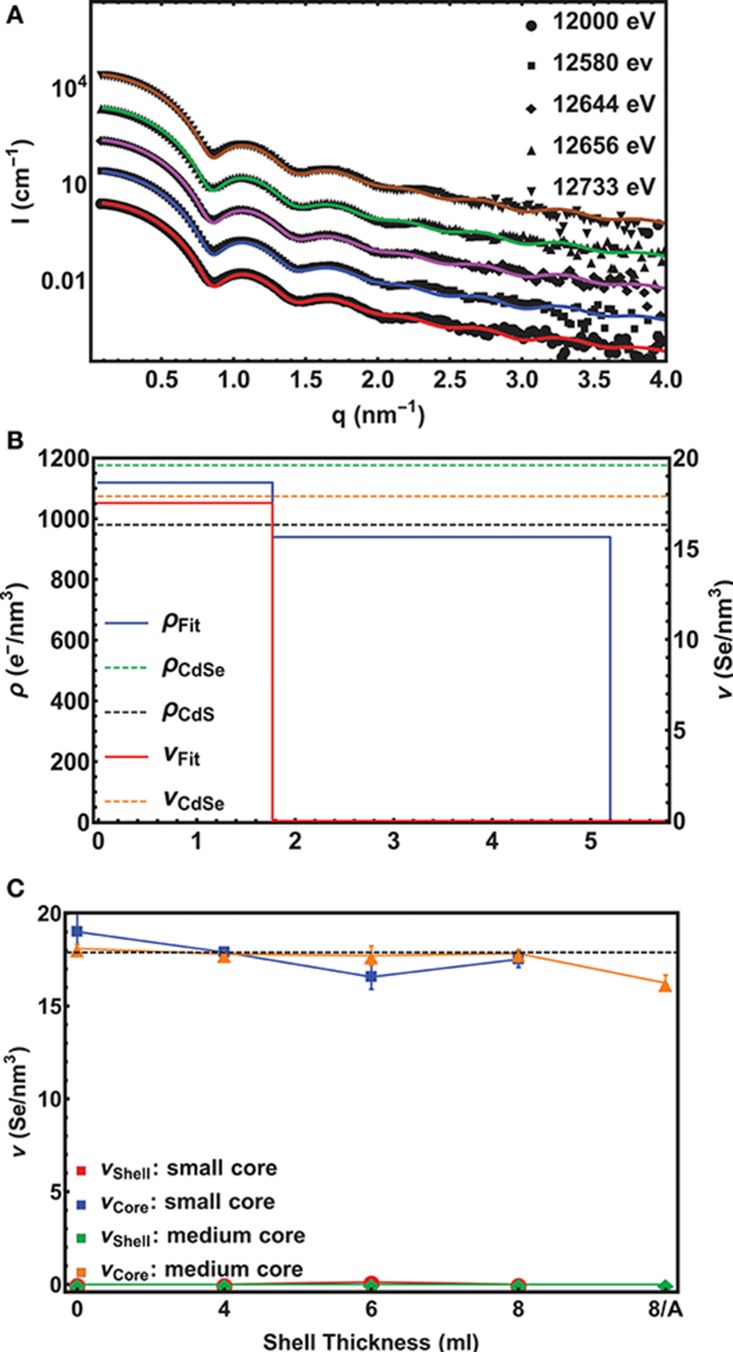
**(A)** Experimental ASAXS curves (symbols) fitted with a unique spherical core/shell model (lines) for the *small* core/shell CdSe/CdS NC-sample with the 8 ML CdS shell. The scattering cross section *I*(*q, E*) plotted over the scattering vector *q* were recorded at 5 different x-ray energies around the Se-K-edge. The scans are shifted vertically for clarity. The lowest curve is plotted in absolute units, whereas the scattering curves above are each scaled by a factor of 10. **(B)** The resulting density profile for the sample in **(A)** s depicted. The Se-density ν (red line) is plotted on the right axis and the total electron density ρ (blue line) on the left axis as a function of the distance from the centre of the sphere. The dashed lines mark the theoretical density values found in bulk materials. **(C)** The Se-concentration ν in the core and the shell as a function of shell thickness is shown. The dashed lines mark the theoretical values for pure CdSe and CdS, respectively. Note that “0” denotes the initial pure CdSe NC and “8/A” the 8 ML thick shell sample after annealing. For the annealed *small* core/shell sample we have no ASAXS data.

In Figure 2A 5 experimental scattering curves are shown together with their model fits for the *small* core/shell NC sample with the 8 ML thick CdS shell on top. (For an example of ASAXS data of the *medium* core series, see Figure S2) Up to four pronounced minima in the SAXS curve directly evidence the narrow size distribution of the NC-ensemble, whereas from the position of the first minimum a fast calculation results in an outer diameter of around 11 nm. The energy dependent effect in the 5 ASAXS curves is visible in the small, but significant shift of the *q*-position of the first minimum in the shape function.

For a quantitative analysis, all 5 curves were fitted with the same unique spherical core/shell model with the following fitting parameter: The inner diameter *D*_*core*_, the shell thickness *t*_*shell*_, the resonant ν_*core, shell*_ and non-resonant ρ_*core, shell*_ electron density differences between the nanocrystal and the solvent, as well as the total size distribution σ of the spherical core/shell NC. The correspondence between data and fit is excellent, smaller deviations are only visible at larger *q*-values, where we also expected to find an influence of a faceted nanocrystal surface, even for an isotropic NC-dimension (Burian et al., 2015, 2018). (In this region, however, the error band of the scattering data is getting larger and the minima of the fit do not fully follow the experimental minima).

The fitting results are summarized in Figure 2B, where the density profile of the *small* CdSe core with the nominal 8 ML CdS shell samples is shown. On the left and right axis the Se-electron density and the total electron (*e*^−^) density as a function of the distance from the center of the spherical fit model is shown. The abrupt drop of the Se-concentration from 17.5±0.7 Se-atoms/nm^3^ to 0 at 1.8±0.05 nm reflects the radius of the CdSe core. The theoretical value of 17.9 Se-atoms/nm^3^ for CdSe in the wurtzite structure is within the error band of our measurement, thus proving a pure CdSe core. Also the value of the total *e*^−^-density with 1120±50 *e*^−^/nm^3^ matches the expected value of 1180 *e*^−^/nm^3^ for CdSe. In the shell region between 1.8 nm and 5.2 nm the *e*^−^-density drops to Δρ_*shell*_ = 940±50 *e*^−^/nm^3^ revealing a pure CdS shell with a theoretical density value of 980 *e*^−^/nm^3^.

This results in a shell thickness *t*_*shell*_ of 3.4±0.05 nm, which is a above the expected value of 2.8 nm for 8 MLs of CdS by assuming an averaged ML-thickness (along all crystallographic directions) of 0.35 nm/ML for bulk CdS. The difference is around 2 ML for the intended 8 and 6 ML shell, for the 4 ML shell we receive only a 1 ML thicker CdS shell as is summarized in Table 1, where all derived core/shell parameters for the *small* and *medium* samples are shown. In contrast, the shell thickness values of the *medium* sized NCs hit the intended ML values within an accuracy of below 0.5 MLs. Only the annealed shell is ~1 ML thicker (see Table 1). We explain the larger CdS shell thickness for the *small* nanocrystals by an inaccuracy in estimation of their concentration. We found, indeed, that on average all three *small* shells before annealing are a factor of 1.2 thicker as compared to the *medium* shells (see Table 1). This indicates that the concentration of CdSe cores has been overestimated by a factor of 1.73 (≈1.2^3^), which is not surprising given that extinction coefficients vary by a factor of 2 for CdSe nanocrystals of such small sizes. Overestimated concentration of cores leads to larger amount of shell precursors per nanocrystal and therefore thicker shell. For the large core/shell NC series this effect will be discussed further below. If we relate the found shell thicknesses with the PL data presented in Figure 1B, we can conclude that shell thicknesses between 6 and 9 MLs give the highest PL quantum yield, especially for the *small* CdSe core samples.

**Table 1:** Listing of the fitting parameters for the spherical core shell model applied to the *small* and *medium* core series.

	**Small Core**				**Medium Core**				
**Series**	**Core**	**4 ML**	**6 ML**	**8 ML**	**Core**	**4 ML**	**6 ML**	**8 ML**	**8 ML Annealed**
*r*_*core*_(*nm*)	1.79	1.77	1.77	1.77	2.09	2.17	2.16	2.17	2.25
*t*_*shell*_(*nm*)	–	1.82	2.73	3.43	–	1.56	2.18	2.94	3.12
*Se_core_*(*n*/*nm*^−3)^	19	18	17	18	18	17	18	18	16
$\rho_{core}\left(e^{-}/nm^{-3}\right)$	1163.53	1111	1149	1119	1168	1085	1220	1165	1156
$\rho_{shell}\left(e^{-}/nm^{-3}\right)$	–	1049	1072	940	–	1034	1056	943	981

*r_core_ denotes the radius of the core, t_shell_ the thickness of the shell. Se_core_ is the atomic density of Selenium in the core, where as ρ_core_ and ρ_shell_ describe the electron densities in the core and the shell respectively*.

Remarkably, in none of the *small* and *medium* core/shell samples a significant amount of Se could be found in the shell. This is also reflected in the constant averaged Se concentration in the cores of 17.7 Se-atoms/nm^3^ at least within ±1 Se-atoms/nm^3^ measurement and analysis accuracy. This is shown in Figure 2C. Even after the 1 h annealing step, no diffusion profile could be deduced from our analysis for the medium sized samples. We tried several diffusion profiles, but all resulted in a larger deviation between fit and experimental data. From this we conclude on a chemical sharp interface between CdSe core and CdS shell. Moreover, the chemical composition as well as the core diameter remains constant during the shell growth with a mean *D*_*core*_ value of 3.6±0.02 nm for the *small* and of 4.4±0.13 nm for the *medium* sized sample series.

## 4. 3D Shape Retrieval From SAXS

**Figure 3:**
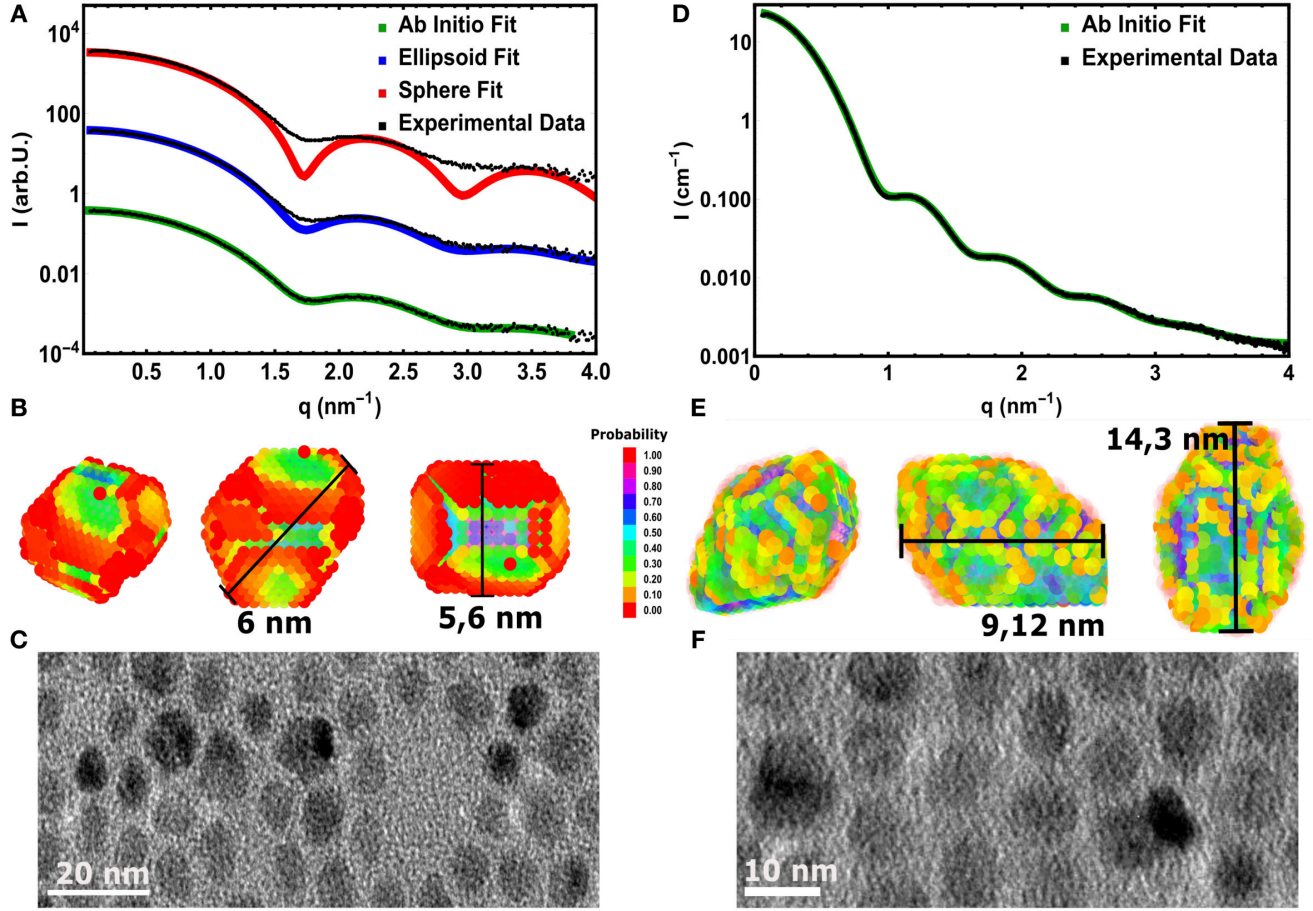
**(A)** Experimental SAXS curve (symbols) of the *large* core CdSe NC-sample fitted with a spherical (red line) and an elliptical (blue lines) polydisperse shape model. The third fitting result (green line) is derived by a assumption free shape retrieval method using freely moveable dummy atoms with 0.5 nm in diameter (see **B**). The line symbolizes the average of 5 independent fitting runs. **(B)** The resulting 3D mean shape as derived from the fits shown in **(A)**. The color represents the probability to find a single dummy atom at the same position after averaging 5 single shapes. The measurement bars show the found longest and shortest axes. **(C)** TEM image of the *large* CdSe cores revealing the surface facets in 2D projections. **(D)** Data (symbols) and fit (line) using the 3D shape retrieval for the *large* core/shell sample with the thickest shell. **(E)** The resulting 3D mean shape as derived from the fits shown in **(D)**, presenting pronounced surface facets and an elliptical shape with an aspect ratio of 1.7. **(F)** 2D TEM images of the *large* core/shell NCs measured in **(D)**.

The same accurate, straightforward ASAXS analysis is not possible for the *large* NC series. The ASAXS fitting procedures give stable and rather unambiguous results for the case of *spherical*, polydisperse core/shell NCs. As shown in Figure 3A, this spherical model can not describe the measured SAXS curve of the *large* CdSe cores sufficiently well. Only the position of the first minimum can be reproduced, but especially around the minima the spherical model deviates drastically from the experimental curve. This position in *q* would correspond to a sphere with around 5.6 nm in diameter. Improved accordance between data and fit up to the 2. minimum at around 3 nm^−1^ in *q* is achieved using a homogeneous ellipsoid of revolution as model, with two long axes and one short axis of 5.6 and 6 nm, respectively (see Figure 3A). The size distribution now is only around 6–8 vol%, but also this fit can not fully reproduce the SAXS curve: The initial slope as well as the depth of the minima cannot be represented satisfactorily by polydisperse ellipsoids of revolution. In both cases -for spheres and ellipsoids- low polydispersity is needed to fit the small *q*-region (<1.5 nm^−1^), but the smeared minima deceptively point to large polydispersity. The aspect ratio in the ellipsoidal model artificially smears the minima, but again the actual minima as well as the initial slope cannot be fit simultaneously.

To gain further information on the shape of the *large* NC series crystals, we employed the ab-initio shape retrieval software DAMMIN (Franke et al., 2017), which has been applied successfully by Burian et al. (2015, 2018) on similar colloidal nanocrystal samples. So-called dummy atoms, arranged randomly in a starting volume, are removed and shifted via simulated annealing until an approximate shape is achieved, which fits the recorded scattering curve excellently (see Figure 3A). Because the resulting dummy atom models are one of several possible configurations, averaging over several, repeating minimization runs is performed. In our case we used five runs, resulting in the faceted real space structure shown in Figure 3B. The interval in *q* used to fit the real space structure was chosen from 0.07 to 3.85 nm^−1^, due to the increased noise in higher *q*-values. When compared to recorded nanocrystal shapes by 2D TEM in Figure 3C, we can see that the CdSe/CdS nanocrystals achieved by hot synthesis show strong faceting of the surface and somewhat irregular crystal shapes. The irregularities of the shape in TEM (see also Figures S3, S4) is due to the nature of the technique, in which only 2 dimensional cuts through individual nanocrystals are observable. Similarly, highly faceted shapes can be reconstructed for any sample of the *large* core series achieving similar qualities of fit.

The same procedure was performed for the *small* and *medium* sized core series, in which no aspect ratio in the range of the large core series, but significant faceting was observed. (Partly visible also in the TEM image shown in Figures S3, S4. High-resolution electron microscopy images were collected on a FEI Talos F200X microscope operated at 200 kV). This is also represented in the fit quality of the *small* core series with the simplified, spherical core shell model. The shape of the initial slope and the first two minima of the NC form factor are quite well reproduced, but higher order minima are not fit satisfactorily. This is mainly due to the additional smearing and slight deviation from the purely spherical shape due to faceting. (This will be topic of a future study).

Although recent studies seem to point to the fact that the probability to find a dummy atom at a certain position as shown in Figure 3B is related to the electron density (Burian et al., 2015) of the particle, we refrain from examining the resulting structures in such a way to differentiate between core and shell. Due to the uncertainty of the shape retrieval method itself, the lowered probability in the outer regions can also be associated with irregularities between the individually retrieved shapes, which are being smeared out by the averaging of the individual results. Consequently one cannot definitively expect the retrieved model in Figure 3B to accurately represent the actual shape of a single nanocrystal. It is rather a mean 3D shape of the whole NC ensemble, including typical features such as the general size, the aspect ratio or the existence of faceted surfaces. The resulting characteristic size parameters of the *large* core series are listed in Table 2.

**Table 2:** The size parameters of the retrieved 3 dimensional dummy atom models for the *large* core series.

**Particle**	**Short axis (nm)**	**Long axis (nm)**	**Aspect ratio (/)**	**Equation diameter (nm)**
Core	5.60	6.00	1.07	5.7
4 ML	6.90	8.50	1.23	7.4
6 ML	9.50	11.20	1.20	10.0
8 ML	7.70	13.40	1.74	9.3
8 ML annealed	9.12	14.30	1.56	10.5

*The equivalent diameter corresponds to a spherical particle with the same volume as the ellipsoids of revolution, with long and short axes*.

Here it can be nicely seen that we started with a nearly isotropic CdSe core with a very small aspect ratio of 1.07, close to our resolution limit. The aspect ratio between short and long axis increases, however, very strongly with increasing shell thickness from about 1.2 up to 1.7 for the thickest shell. After annealing the shell is still slightly growing but also shows indications of surface restructuring, which is reflected in the lowered aspect ratio of 1.6 as can be seen in Figure 3E. We can retrieve now the shell thickness values along the short and long axis by subtracting the core axes from the total diameter values. In general, for all samples both shell directions are getting thicker with increasing ML coverage, but the increase is not so homogeneous with growth time as observed for the *small* and *medium* sized NCs. From the nominal 6–8 ML growth step the short shell thickness actually decreases. Because this is not possible in systems which are not precursor deficient, on reason could be the limited amount of structural information retrievable from scattering techniques. Because the overall number of Shannon Channels is set at 8 for this sample, we probably cannot fully retrieve the actual shape in full detail due the strong anisotropy and faceting of the nanocrystals. What one gets from averaging several shapes retrieved by ab-initio approaches like DAMMIN is a most likely representation, featuring the most dominant characteristics of the shape of the sample under investigation. This is shown in Figures 3B,C,E,F, where facets, both in the TEM images and the reconstructed shapes, can be seen. The overall size and structure is mostly determined by the minima of the SAXS-patterns in Figures 3A,D, whereas the smeared nature of the minima indicates faceting and anisotropy in the shape. However, one clear overall trend can be observed: The shell growth along the short axis is clearly below the intended ML thickness values, whereas along the long axes the *t*_*shell*_ values are up to 2 ML thicker as intended. The results of the 3D shape analysis allows the conclusion that some of the core facets enables better CdS growth than others. These pronounced surface facets are certainly induced by the crystal structure of the NCs, but from the SAXS based 3D shape analysis alone we can not conclude on the crystallographic direction of the crystal faces. Taking the detailed TEM studies on CdSe/CdS NCs into account one would expect to find the anistropy axes axis parallel the hexagonal *a*− and *c*-axis Peng et al. (1997).

The combination of both techniques -ASAXS and 3D shape retrieval- delivers many parameters of the internal structure of the core/shell NCs. From ASAXS we get reliable information on the internal chemical core/shell profile and from the shape retrieval method on the outer NC shape. Although we were only able to get full information on the chemical composition of the *small* and *medium* core series, both of which exhibit a chemically sharp boundary between core and shell, we are confident to assume the same for the *large* core series. So two open questions remain: First, what is the physical reason that the *large* NCs develop the revealed pronounced elliptical shape and large surface facets? The facet formation should be driven by the *wurtzite* crystal structure of CdSe and CdS. This leads to the second question: Which is the crystallographic direction of the short and long axis? These questions may be answered by analysing the powder x-ray diffraction data (XRD) measured simultaneously with the ASAXS data by recording the wide angle x-ray scattering (WAXS) pattern in transmission geometry.

## 5. Wide Angle Scattering Analysis

**Figure 4:**
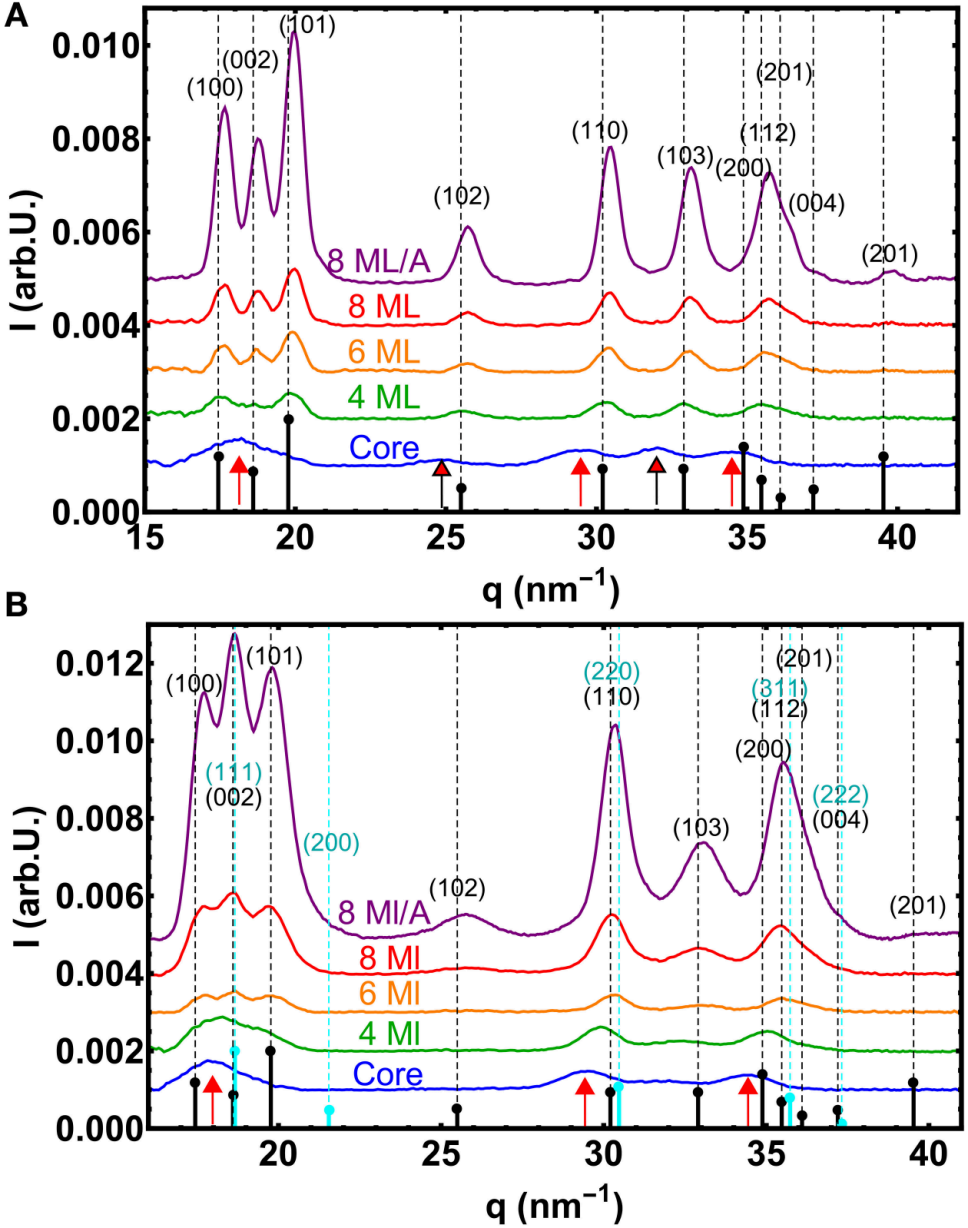
**(A)** WAXS curves of the *small* core/shell NC series. The vertical dashed lines mark the theoretical positions of the Bragg peaks for unstrained bulk CdS in the hexagonal *wurtzite* phase. The theoretical relative intensities are symbolized by small black sticks, whereas the red arrows mark the theoretical positions for *wurtzite* CdSe. **(B)** The same for the *large* core/shell NC series, but now also with the marked positions and intensities the for cubic *zincblende* CdS phase (cyan). Here the red arrows mark the positions of cubic *zincblende* CdSe.

In Figure 4A the WAXS data of the whole *small* NC series are shown. The dashed lines mark the theoretical Bragg peak positions for bulk CdS (*a* = 4.14 Å, *c* = 6.75 Å) as well as the expected intensity ratios (black sticks). The lowest diffraction peaks has clearly the small core CdSe NCs. With increasing shell thickness and thus increasing total diameter the peak intensity increases, as the diffracted intensity is proportional to the particle volume square. (The total XRD intensity depends also on the NC concentration within the solvent toluene. The annealed sample depict the highest NC concentration). Furthermore the width of the peaks decreases with increasing NC-diameter as is expected for an increasing crystalline particle sizes. From fitting the powder diffraction data with Gaussian peaks on a splined background, we derive the full width at half maximum FWHM (Δ*q*) of the Bragg peaks along specific crystallographic directions. From Δ*q* one can calculate the crystallite size *D*_*hkl*_ using the Scherrer equation with *D*_*hkl*_ = *F*·2π/Δ*q*, where *F* is a constant with values close to 1, but depends on crystallographic direction or particles shape (Als-Nielsen and McMorrow, 2011).

The positions of the peaks are determined by the crystalline structure and the chemical composition, but also by all influences that changes the *d*-spacing within the atomic lattice, such as internal strains or external applied pressures. As an overall trend one can observe that the peaks shift from the initial CdSe position (marked by red arrows) toward the CdS positions, as the CdS shell forms the major part of the core/shell scattering volume when the shell thickness reaches 4 ML (see Figure 4A). (The core/shell volume ratio for the 4 ML shell is around 1/7 resulting in an about 1/50 XRD intensity ratio).

For the CdS shells above 4 ML thickness all peak positions are, however, at larger *q*-positions as the theoretical values. Larger *q*-values mean smaller lattice spacings and thus the nanocrystal structure is under *compressive* strain. As the peak shift along all crystallographic directions is roughly the same, a hydrosatic pressure caused by a surface relaxation due to the high surface/volume ratio for small sized NCs can be the reason. A hydrostatic decrease in lattice spacing was also found for other NCs with comparable elastic constants, like ZnS, but only for diameters below 5 nm (Rath et al., 2008). The total diameter of our CdSe/CdS NCs would exceed this limits for the thicker shells, but it was reported especially for 2 nm CdS shells around PbS cores that also the shell alone can be compressed (Lechner et al., 2014).

Also a new polytype of the CdS crystal structure or even a non-periodic lattice within a closed packed nanocrystal can result in shifted or vanishing peaks as shown for CdS-NCs (Rempel and Magerl, 2010). In our measurements two pronounced peak triples, the (100)-(002)-(101) and the (110)-(103)-(112) triple, are visible that are characteristic for the hexagonal *wurtzite* structure as is shown in Figure 4A. For the smallest core sample the first peak triple cannot be easily resolved due to overlapping of three broad peaks. (Within the second triple the (112) peak consists in fact again of three individual peaks). Furthermore, the peak intensity ratios of the first triple nicely follow the theoretical values for *wurtzite* CdSe. When we additionally analyse now the width Δ*q* of all visible diffraction peaks and calculate the total crystalline diameter *D*_*hkl*_, we derive an rather isotropic size for the whole *small* core/shell series (see Table 3), with rather small deviations in Δ*q*. (Here we neglect in a first approximation the peak broadening due to stacking faults and other crystal defects. As we calculate mainly the chord length of “hollow” CdS spheres we use the Scherrer constant *F* = 1). The largest difference in size we found not along the hexagonal [100] and [001] directions, but between “straight” and “tilted” surface facets. The *straight* planes are either fully parallel to the hexagonal *a*- or *c*-axis. The corresponding size *D*_||, ⊥_ is related to the crystalline particle dimension that are terminated by the {*h*00}, {00*l*} and {*hh*0} lattice planes. The *D*_||, ⊥_ values are derived by averaging the sizes obtained by the (100), (002), and (110) peak widths. The *tilted* plane values *D*_*tilt*_ are related to planes containing *a*- and *c*-axis components and thus having {*h*0*l*} Miller indices. The *D*_*tilt*_ values are calculated from the widths of the (101), (102), and (103) peaks. All absolute *D*_*hkl*_ values are always smaller as the diameters derived by ASAXS. This can be related to additional peak broadening by lattice defects or by not fully perfect crystalline top most surface layers (see Table 3). For spherical core/shell NCs, however, we have to take an additional effect into account. For thick CdS shells, where the CdSe core can be neglected, the width of the CdS peaks originate from hollow CdS sphere with a certain shell thickness. With XRD we hence probe rather the*weighted chord lenght* (Gille, 2000) of the CdS shell instead of directly the shell thickness or even the total NC diameter. For spherical NCs the shell thickness or diameter can be calculated, but for strongly anisotropic and faceted shapes absolute structural parameter can not be easily deduced.

**Table 3:** The crystallite sizes of the *small* and *large* core/shell series as derived from the analysis of the WAXS pattern shown in Figure 4.

	***D*_||,⊥_ (nm)**	***D*_*tilt*_ (nm)**	**Aspect ratio**
**SMALL CORE**			
Core	3.3	–	1
4 ML	6.2	6	1.05
6 ML	6.9	7	0.99
8 ML	8.1	7.3	1.10
8 ML/A	7.6	7.4	1.03
**LARGE CORE**			
Core	4.5	–	–
4 ML	5.2	–	–
6 ML	6.3	5.3	1.20
8 ML	7.9	5.6	1.42
8 ML/A	8.4	5.8	1.46

*D_||, ⊥_ denoted the crystallite size parallel to the straight planes, D_tilt_ parallel to the tilted planes. The relative size accuracy is ±0.1 nm*.

For a first comparison between SAXS and WAXS data we calculate the chord length values for the different CdS-shell thicknesses for the *small* and *large* series. For the *large* core/shell NCs we use the derived spherical equivalent diameters as listed in Table 2. All these value are compared to the derived crystallite sizes as listed in Table S1 (see Supplementary Material) and proves a really good accordance between overall dimensions and crystallite sizes.

We can also calculate an crystalline aspect ratio between *D*_||, ⊥_ and *D*_*tilt*_ as listed in Table 3. For the *small* CdSe core sample we could not derive all peak-width values due to overlapping peaks. But where it can be calculated we derive an isotropic crystal size of around 3.3 nm. But also all other aspect ratio values are below 1.1, which is close to our resolution limit, indicating an isotropic core/shell shape.

To summarize the XRD analysis of the *small* NC series, we can conclude that both, CdSe core and CdS shell, keep their hexagonal *wurtzite* crystal structure, but the thicker shell samples depict an compressive strain between 0.6 and 1%. The found crystallite size reflect again the rather isotropic shape of these NCs as already evidenced independently by the ASAXS analysis (compare Table 1). An analysis of the *medium* core series was also conducted, but it is not necessary to illustrate the effects discussed in this paper. The corresponding WAXS pattern is depicted in the Supplementary Material (see Figure S4).

A rather different picture results from the analysis of the WAXS data of the *large* core/shell series as shown in Figure 4B. The first directly visible effect is the changed intensity ratios of the first peak triple, the (100)-(002)-(101) peaks: Here the middle peak—the hexagonal (002) peak—is the strongest peak of the three, clearly visible for the samples with CdS shells. The first triple is not visible for the *large* core CdSe sample, but in contrast the middle peak of the second triple—the (103)–vanished. Also the (102) peak around 25 nm^−1^ is no longer visible. This can be explained by the fact that the CdSe core now has a changed crystal structure, i.e., the cubic *zincblende* structure ($F\bar{4}3m$), with the equivalent cubic lattice constant of 5.832 Å. The unstrained *q*-positions for the new CdSe core phase are additionally marked in Figure 4B by the three red arrows, whereas the positions and relative intensities of cubic *zincblende* CdS are marked by cyan dashed lines and sticks.

This XRD pattern, missing *wurtzite* (102) and (103) peaks, but increased (002), is also visible for the 4 and 6 ML shell sample. For the case of the 4 ML CdS sample this could be explained by a cubic *zincblende* CdSe core with an *wurtzite* CdS on top, because for this shell thickness the scattering volumes of core and shell are comparable. By reaching shell thicknesses equal and larger of 6 ML the shell-volume clearly exceeds the core-volume and thus the main diffraction contributions originates from the shell's crystal structure. Indeed, the *wurtzite* (103) peak appears again, but really faintly making a fitting rather ambiguous. Within the first peak triple, however, the central (002) peak is still the strongest peak. The same behavior can be observed for the thickest shell, where additionally the (102) peak is back again and now also the (103) peak is clearly visible indicating a larger *wurtzite* phase fraction within the CdS shell. The intensity ratios of the whole XRD pattern, however, can be only explained by a *considerable* amount of a cubic *zincblende* phase fraction within a hexagonal CdS shell. The additionally cubic (111) Bragg peak at the position of the hexagonal (002) causes the increased central peak by adding up both peak intensities. For spherical core/shell particles it would be possible to simulate the diffraction patterns for the different crystal structures and hence determine quantitative values of the amount of *zincblende* phase fraction within core and shell as shown (e.g., in Lechner et al., 2014). For the strong anisotropic and faceted NC-shape these simulations are not so straight forward and will be topic of an upcoming study.

### 5.1. WAXS-SAXS-PL

For further insight we analyse the FWHM of the peaks to derive the crystallite sizes *D*_||, ⊥_ and *D*_*tilt*_ perpendicular to the *straight* and *tilted* planes and thus calculate the aspect ratio between these directions. This analysis is, however, not fully possible for the *large* CdSe core sample and for the shells up to 6 MLs, because especially the {*h*0*l*} peaks are not visible, or can not be resolved within merged triple peaks (see Figure 4). For the 6 ML sample the three straight peaks and only one tilted, the (101) could be resolved. The derived chord length values (listed in Table 3) clearly increase with growing shell thickness and are only around 10% below the values as estimated from the core/shell parameters derived from SAXS.

For the two 8 ML large core/shell sample we can analyse three *straight* peaks ({*h*00}, {*hh*0}, {00*l*}) and three tilted peaks ({*h*0*l*}).The 8 ML annealed sample show clearly increased *D*_*hkl*_ values in both directions with respect to the 8 ML shell sample without annealing. This indicates that during annealing the crystalline shell part growths, or that the shell's crystal quality is improved also resulting in a narrower peak. For the 6 ML and both 8 ML samples the found crystalline aspect ratio values are listed in Table 3 and ranges from 1.2 to 1.5. These values are very close to the values derived from the SAXS based 3D shape analysis, where we are not sensitive to the crystal structure (see Table 2).

The accordance between these two independent data sets allow now to relate the found long NC axes to the *straight* crystal planes ({*h*00}, {00*l*}, and {*hh*0}) and the short axes to the *tilted* planes ({*h*0*l*}).

This result has some physical evidence as the CdS shell growth on the tilted CdSe planes is not so efficient as on straight planes, because for the shell growth on planes with {*h*0*l*} Miller indices additional monolayer steps has to be introduced. As this effect is not observed for the *small* NC series and only partly for the *medium* sized series (not discussed in this paper), the presented explanation is not sufficient for explaining the derived experimentally derived NC-parameters.

We found, however, already a mixed cubic/hexagonal phase fraction within the large CdSe core that result in increased crystal defects within specific surface facets. These defects will result again in increased stacking faults within the subsequent CdS shell and may enable more effective shell growth parallel to the *straight* crystal planes. An increased number of lattice defects or stacking faults, however, should result in strain relaxation within the CdS shell (Wickham et al., 2000; Zaziski et al., 2004). This is indeed observed in Figure 4, where NCs of the *large* series with the thicker shells exhibit less compressive strain (below 0.01%) as compared to the *small* NC series.

For further insight, the overall mechanism of deformation for hexagonal systems has to be discussed shortly. The overall criterion for the type of deformation in hexagonal lattices is the *c*/*a* ratio, which is slightly above 1.63 for CdS. For any hexagonal material, deformation for *c*/*a* ratios below 1.63 is found on basal, pyramidal and prismatic lattice planes, above 1.63 only in basal planes and above 1.73 through conformal movement via twinning (Gottstein, 2013). Twinning can also be found in materials with *c*/*a* ratios close to 1.63, but only in the context of polycrystalline materials. In these cases at least 5 glide systems need to be active to ensure deformation. For crystals with free surfaces and a *c*/*a* ratio at 1.63, the three glide systems provided by glide on the basal plane are enough to ensure sufficient deformation to relieve strain, hence we do not expect deformation by twinning.

We expect so called Shockley dislocations, i.e., two partial dislocations (Gottstein, 2013), to form on the basal plane to reduce the line energy connected to the length of the associated burgers vector (Berghezan et al., 1961). These dislocations extend through the crystal on the (001) plane, which coincides with the (111) plane of the fcc *zincblende* lattice upon dislocation movement and consequent change in stacking order from *ABAB* to *ABCA* (Berghezan et al., 1961). Consequently, we can expect crystals, which are able to release stresses via dislocation glide forming stacking faults perpendicular to the basal plane, changing the stacking order locally from hexagonal to face centered cubic. With these observations we think we can explain the fact that the cores exhibit increasingly *zincblende* structure with increasing core size. In the case of large core particles, the overall structure seems to be *zincblende* for the core and increasingly more *wurtzite* phase seems to exist in the nanocrystals with increasing shell thickness. Similar mechanisms can be responsible for the phase transition from *zincblende* to *wurtzite*. The dominant glide system in *fcc* materials are directions situated on the (111) plane, on which the Burgers vector can split into two partial dislocations to minimize its line energy (Gottstein, 2013). The stacking is then also changed, but this time from *ABCABC* to *ABCBCA*, meaning we get small but extended *wurtzite* domains inside the *zincblende* structure due to the fact that the respective (111) and (001) planes coincide. Indeed, we see some streak-like features in some nanocrystals of the *large* core series with 8 ML shell and annealing and can distinguish stacking faults (see Figure S3). These defects in the CdS shell should be reflected in a reduced PL emission due to a partial extension of the exciton wavefunction into the shell material (Reiss et al., 2009). Indeed, the *large* core/shell NCs with pronounced shape anisotropy and surface facets show an at least 20% lower PL-QY as compared to the *small* and *medium* sized samples (see Figure 1C). A possible reason for this pronounced difference might simply be the size difference between both series. The *large* core size of 5.8 nm simply offers enough space for creating dislocations to compensate strain (Peierls, 1940; Demortiere et al., 2018) and trigger the phase transition from *wurtzite* to *zincblende*. Coherently, the *small* core series exhibits a single, but strained, *wurtzite* structure in contrast to the predominately *zincblende* phase fraction within the *large* core series.

### 5.2. Conclusion and Outlook

A more detailed analysis of all WAXS data in combination with atomistic simulations of the XRD patterns in a future study will give more quantitative results on, e.g., dislocation densities along specific crystallographic directions. This will allow to follow the transition from smaller, isotropic NCs with a single hexagonal *wurtzite* crystal structure to larger anisotropic NCs with a mixed hexagonal and cubic *zincblende* phase, especially by analysing the data of the *medium* sized CdSe/CdS samples.

The size change from the initial *small* CdSe core from 3.6 nm, over the *medium* sized core with 4.4 nm to the *large* core of 5.7 nm corresponds to *only* 3 ML more CdSe on top of the *small* core. The combined (A)SAXS/WAXS measurements and analyses allow to resolve such small changes in the NC dimension on a large ensemble of NCs that is needed in optical applications. It clearly shows the drastic change to an anisotropic shape, when the a bit larger core exhibit a mixed crystal phase fraction. Annealing at 310°C does not affect the chemical sharp CdSe/CdS core shell interface and slightly reduces the large aspect ratio for the largest core/shell NCs derived from SAXS, but not the crystallite aspect ratio as derived from WAXS. This can be explained by an exclusively reordering of the amorphous (or partly crystalline) top most shell layer not affecting the main part of the CdS shell. This would also explain why the photoluminescence output after annealing is not significantly increased.

When we summarize all structural and optical derived NC parameters presented in this study on CdSe/CdS, we can conclude the following: An initial difference in the CdSe core dimension of only 2 MLs finally results in a 20% decreased PL output. Especially for high efficiency CdSe/CdS NCs with QYs above 90% (Chen et al., 2013) such changes has to be understood and—finally—has to be controlled. That means during synthesis of optical active NCs, the NCs' size has to be controlled in monolayer accuracy *and* that the applied characterization techniques has to deliver an even better accuracy in the range of atomic resolution. This resolution in 3D should be obtained for both, the chemical and crystalline profile of core/shell NCs.

## Data Availability Statement

Raw data were generated at the large-scale synchrotron radiation facility ESRF. Processed data are available from the corresponding author RTL on request.

## Author Contributions

LL, RTL, MS, and PB conducted the anomalous small angle and wide angle x-ray scattering (ASAXS/WAXS) experiments. LL and RTL performed the ASAXS/WAXS data evaluation. DND and MVK synthesized and prepared samples and performed TEM as well as PL measurements. LL and RTL wrote the manuscript, RTL envisioned, planed and supervised the research.

### Conflict of Interest Statement

The authors declare that the research was conducted in the absence of any commercial or financial relationships that could be construed as a potential conflict of interest.
