# Supplementary material for: Impact of Crystal Structure and Particles Shape on the Photoluminescence Intensity of CdSe/CdS Core/Shell Nanocrystals
Source: Front Chem. 2019 Jan 22;6:672. doi: 10.3389/fchem.2018.00672 (PMC6350456; doi:10.3389/fchem.2018.00672)
Supplement: Supplementary file 1 [file Data_Sheet_1.PDF]

Supplementary Material: Impact of crystal structure and particles shape on the photoluminescence intensity of CdSe/CdS core/shell nanocrystals

1 SUPPLEMENTARY TABLES AND FIGURES

1.1 Equations

The core shell form factor used in this work to fit the ASAXS data is

$$F(q) = \rho_1 V_{r_1} \frac{3(sin(qr_1) - qr_1 cos(qr_1))}{(qr_1)^3} - (\rho_1 - \rho_2) V_{r_2} \frac{3(sin(qr_2) - qr_2 cos(qr_2))}{(qr_2)^3} \quad \text{(S1)}$$

with r_1 and r_2 being the outer shell and core radius respectively and ρ_1 and ρ_2 describing the scattering densities of the shell and core. How the single contributions due to the contrast variation were fitted can be found in Ref. Lechner et al 2014 in the paper.

1.2 Tables

The ratio of the mean size derived from SAXS from the *small* Core series is shown in table S1:

Table S1. The crystallite sizes of the *small* core/shell series as derived from the analysis of the WAXS pattern shown in figure 4 in the main paper. The mean of $D_{||,\perp}$ and D_{tilt} ,denoting the crystallite size perpendicular and parallel to the *straight* planes as outlined in the main text, is calculated. This is compared to a mean chord length of a hollow sphere. The outer and inner diameter of the core shell particles from the ASAXS measurements are taken as input for the chord length calculation. The results are then compared and seem to compare favourably for the spherical *small* cores series.

Small Core	D-WAXS (nm)	D-SAXS (nm)	Chord Length (nm)	WAXS / Chord Length
Core	3.3	4.58	3.04	1.09
4 ML	6.06	7.18	4.89	1.24
6 ML	6.95	9	6.67	1.06
8 ML	7.75	10.4	7.81	0.99
8 ML/A	7.5	not available	/	/

The ratio of the mean size derived from SAXS from the *large* Core series is shown in table S2:

Table S2. The crystallite sizes of the *small* core/shell series as derived from the analysis of the WAXS pattern shown in figure 4 in the main paper. The mean of $D_{||,\perp}$ and D_{tilt} ,denoting the crystallite size perpendicular and parallel to the *straight* planes as outlined in the main text, is calculated. This is compared to a mean chord length of a hollow sphere. The outer and inner diameter of the core shell particles from the ASAXS measurements are taken as input for the chord length calculation. the results are then compared and seem to compare favourably, eventhough the *large* core series samples exhibit a largely elliptical shape.

Large Core	D-WAXS (nm)	D-SAXS (nm)	Chord Length (nm)	WAXS / Chord Length
Core	4.5	5.73	4.86	0.93
4 ML	5.22	7.39	5.4	0.97
6 ML	5.785	10.05	7.75	0.75
8 ML	6.735	9.31	7.11	0.95
8 ML/A	7.105	10.54	8.13	0.88

1.3 Figures

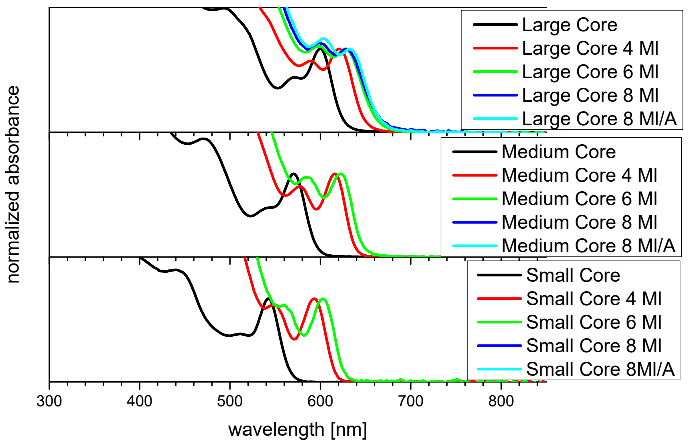

Figure S1. The normalized PL data is depicted for all samples in this work. At the very top the spectra for the *small* core series are depicted, in the middle the *medium* core spectra are shown and at the very bottom the *large* core series PL is displayed.

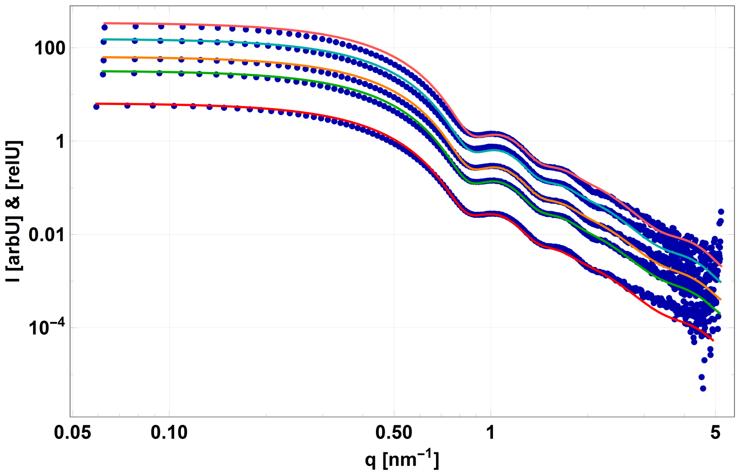

Figure S2. Depiction of the spherical core shell fit for the *medium* core 8MI/A sample at five different X-ray energies. The lowest pattern is shown in absolute units, whereas all others are shifted by a constant factor. Due to increasing faceting and slight anisotropy (maximum aspect ratio around 1.08) of the nanocrystals, the low q-region is deviating from the fit-function. Probably due to the smaller size, the surface faceting is not as pronounced as in the *large* core series and fitting by a spherical core-shell model seems to be still sufficient. The overall sizes of the *medium* core series can be looked up in table 1 in the main work of this publication.

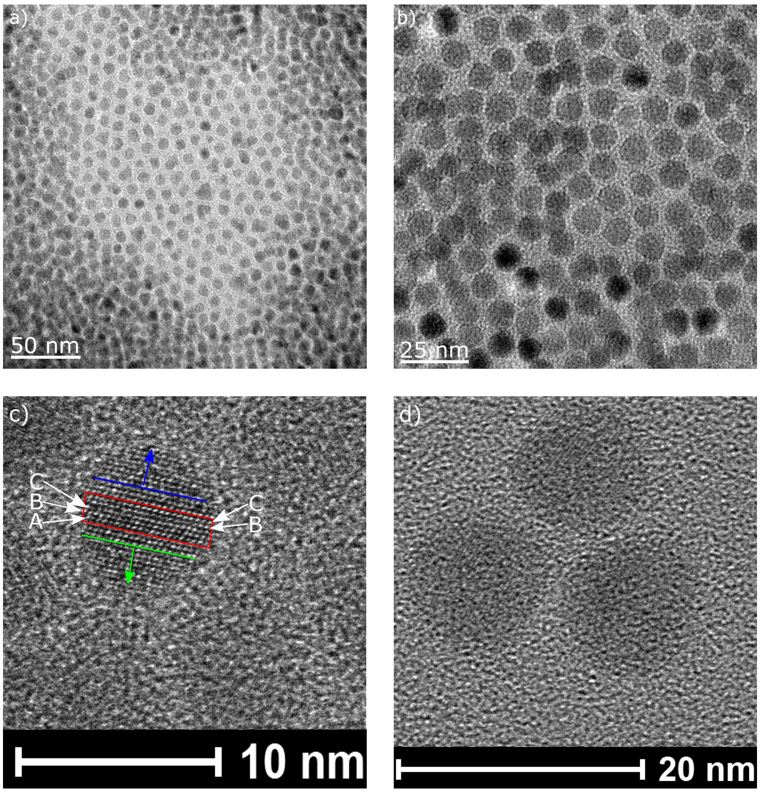

Figure S3. TEM images of the *large* core (a) and *small* core (b) series, 8ML/A nanocrystals. As is immediately evident, the *large* core nanocrystals exhibit larger asymmetry as the *small* core series crystals. It can also be deduced, that there may be a preferential alignment of the *large* core nanocrystals, as a few are not totally aligned, which leads to faults in the closest packing visible in a. In contrast, nanocrystals in b seem more regular in shape and overall size. In (c) we show a HRTEM picture of a large core 8ML/A crystal. The red box highlites the are where a stacking fault is clearly observable on the (110) facet, with the stacking order pointed out by the letters. First the stacking order describes the Zincblende structure, then a singular stacking fault is visible which locally changes the order to Wurtzite. The green and blue lines indicate regions, from which on different facetes of the nanocrystal are depicted, which can be deduced by the different ordering of the atoms. The quadractic arrangement of the atoms suggests, if we assume a predominantly Zincblende structure, a (100) type facet on both sides indicated by the colored arrowes. (d) Shows the small core 8 ML/A particles, which are clearly showing a round shape.

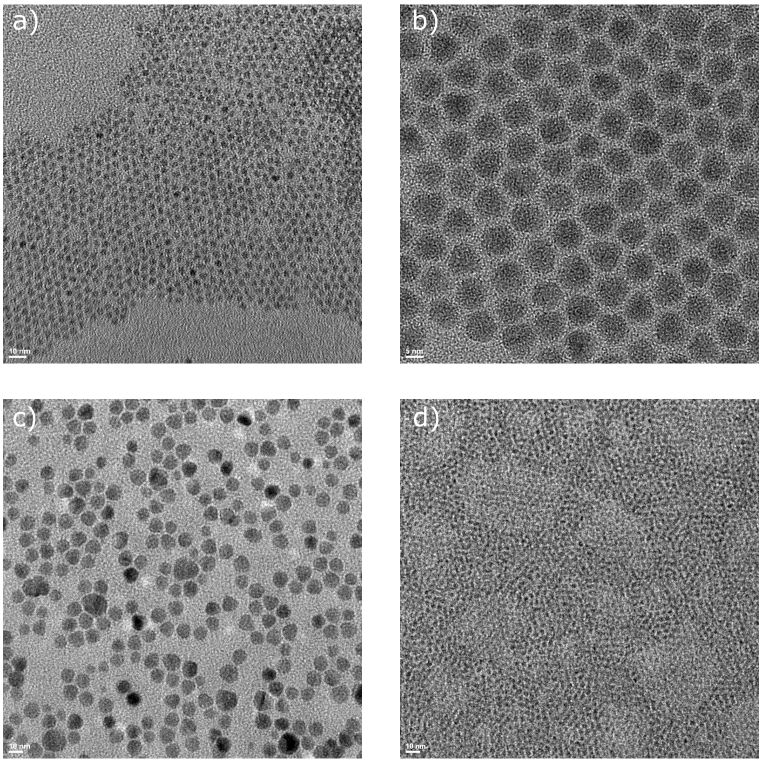

Figure S4. TEM images of the *small* core (a) and *medium* core (b,c,d) series. In (a) we show a low resolution TEM image of the *small* CdSe cores whereas in (b,c,d) we show low resolution TEM images of the *medium* core 6 ML, 8ML/A and CdSe core samples of the *medium* core series.

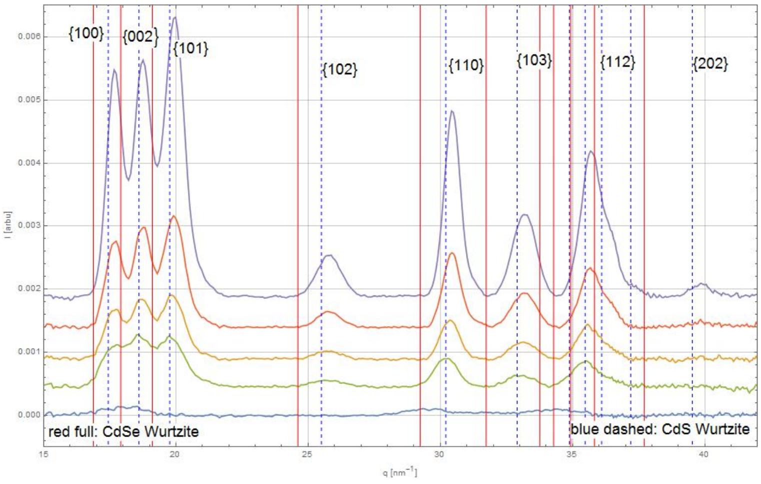

Figure S5. The wide angle X-ray scattering patterns are shown for the medium core series. Because the peak intensities don't match with the theoretical bulk values or the wide angle scattering pattern of the *small* core series (Fig. 4.a in paper), we can conclude that a mixture of zincblende and wurtzite structure is present in the nanocrystals. When comparing this pattern to the *large* core series depicted in Fig. 4.b, we can see that the overall zincblende fraction is significantly less than in the *large* core series.
